# Strategies to Maximize the Involvement of Undergraduates in Publishable Research at an R2 University

**DOI:** 10.3389/fpsyg.2019.00214

**Published:** 2019-02-12

**Authors:** Gary L. Dunbar

**Affiliations:** Department of Psychology, Central Michigan University, Mount Pleasant, MI, United States

**Keywords:** peer-mentoring, team-based research, R2 university, graduate-student mentoring, interdisciplinary programs

## Introduction

In 1987, I was hired by the Department of Psychology at Central Michigan University (CMU) with the primarily responsibility of teaching courses in physiological psychology, which were required for accreditation of our Doctor of Psychology (Psy.D.) program, the only doctoral program at CMU at that time. I was given $3,500 start-up and lab space which consisted of a 12′ X 24′ storage room and a similarly sized retrofitted classroom to house rodents. However, by refurbishing discarded equipment from companies, like Dow Chemical and Dow Corning, and building our own rat mazes, my students and I were able to put together a functioning behavioral neuroscience laboratory that allowed students to engage in hands-on, inquiry-based research (Dunbar, 1998). Although CMU had a M.A. program in experimental psychology, nearly all the department-supported graduate assistantships went to the Psy.D. program, so my lab mainly consisted of undergraduates, with two self-funded M.A. students. With the help of two equipment and course improvement grants garnered through the National Science Foundation and by partnering with a Michigan-based pharmaceutical company (Upjohn), we were soon able to conduct publishable research, which included undergraduate students. Our undergraduate research blossomed with the infusion of innovative ideas and interactions with members of a new organization, the Faculty for Undergraduate Neuroscience (FUN).

## The FUN Begins

At the annual meeting of the Society for Neuroscience in 1991, a group of neuroscience faculty members with a shared passion for teaching and involving undergraduates in research formed a new organization, the Faculty for Undergraduate Neuroscience (FUN) (Ramirez and Normansell, 2003; Dunbar and Symonds, 2018). The networking that FUN provided had a major impact on my ability to engage undergraduates in publishable research. Ideas of peer-mentoring among students (Mickley et al., 2003) and helpful tips on writing successful grants translated into accommodating more students in my lab and into writing a successful R15 grant from the National Institutes of Health.

In 1995, the first FUN workshop took place at Davidson College (Ramirez and Normansell, 2003; Hardwick et al., 2006), which initiated a series of national blueprints for a core undergraduate neuroscience curriculum (Ramirez, 1997; Ramirez et al., 1998; Wiertelak and Ramirez, 2008; Kerchner et al., 2012; Wiertelak et al., 2018). In addition, there were sessions on neuroscience pedagogy and on ways to augment publishable research for undergraduates (Dunbar, 1998, 2001; Mickley et al., 2003). The Davidson workshop provided the impetus for establishing an interdisciplinary program in neuroscience at CMU and to develop the first undergraduate neuroscience major in Michigan in 1999 (Dunbar, 2015). We were able to do this by patching together a curriculum of existing courses from biology, chemistry, psychology, and health sciences, which minimized the costs. In addition, faculty who served the program did this as a voluntary overload, in addition to their contractual obligations to their home departments. However, we later found that because the solid academic structure of a department was lacking in our interdisciplinary program we were particularly vulnerable to the machinations of inter-departmental politics that gave us no voice or seat at critical decision-making tables. Nonetheless, the establishment of the new program in neuroscience provided a huge boost to our research programs and provided a foundation that would lead to a significant increase in the number of publications involving our undergraduates (Dunbar, 2015).

## FUN Lessons Learned

By employing some of the critical lessons received from FUN workshops (which occur every 3 years) and publications, such as FUN newsletters and the Journal of Undergraduate Neuroscience Education (Lom, 2002; Dunbar et al., 2009), our neuroscience program at CMU flourished and garnered attention from the CMU administration, which soon translated into internal support for our program.

The key to our success has been the implementation of a team-based, peer-mentor approach, whereby experienced, more senior undergraduates serve as research mentors to less-experienced undergraduates who assist the advanced students with designated parts of a research project for which they are responsible (Mickley et al., 2003). These less experienced undergraduates work with their mentors to help complete a larger study, such as a senior thesis of the advanced student, or, in most cases, some component of a larger, more comprehensive project which is overseen by the faculty member or graduate student.

All students in my lab are required to write a proposal that includes a new aspect of the research being formulated (such as an additional behavioral test) that would enhance the scope of the overall project. This allows students the opportunity to provide intellectual contributions and ensures that they are conceptually connected with their portion of the work and to the overall project. Students present their specific proposal at lab meetings where they are assessed in a manner that simulates how peer-reviewed panels of granting agencies evaluate proposals. After receiving feedback on their proposals, the students refine their ideas and present them at subsequent meetings until they convince their fellow team members and others in the lab that one of their ideas should be incorporated as a part of the overall study.

The advanced undergraduates and all the graduate students in my lab are then required to write up a grant proposal over their portion of the overall project and submit it for possible funding to either our internal, university-wide student grant program or to external granting agencies. These students and their mentees then help collect and analyze data, as well as provide drafts of their portion of the manuscript. Finally, the students review the completed manuscript and provide suggested edits prior to submission and during the re-submission process. This step is critical for honing the writing skills of the students.

At first, we started with 2–3 teams of 4–5 students per team, and though less than one-third of the projects we conducted resulted in a publishable outcome, students learned the rudiments of how to conceptualize and conduct a research project and how to present their results, including giving oral presentations and producing written manuscripts. Over time, the quality of the manuscripts continued to improve, resulting in an increase in the number of publications that involved undergraduates from my lab (Dunbar, 2015). Much of the improvement in the quantity and quality of published articles from my lab should be credited to the growing number of graduate students working in my lab, an advantage afforded at an R2 university. However, the growth of our program and the greater emphasis on garnering more grant money made it increasingly difficult to focus on the student-centered research that elevated the stature of our lab and program.

## R2 Advantages and Disadvantages

Although there are several advantages that my colleagues at smaller, liberal arts colleges have in terms of being able to forge interdisciplinary programs with less complications than many of us encounter at larger universities (González, 2001), there are some major advantages to being at anR2 institution. One of these is to utilize graduate students in a team-centered approach to student research. In 2008, we successfully launched an M.S. and Ph.D. program in neuroscience at CMU that was designed to bolster our undergraduate program by increasing the number of graduate students to help mentor our undergraduates. Specifically, we utilized some of the ideas promulgated at the FUN workshops, especially utilizing teams of undergraduates with upperclassmen (and, for our program, graduate students) providing mentorship to the lower classmen.

The major advantage of successfully utilizing our graduate students as mentors for the undergraduates became apparent by 2012, when senior graduate students began supervising 2–3 of the undergraduate teams. These senior graduate students formulated parallel projects that, when combined with experiments of the undergraduate team, provided for stronger research findings, which eventually found their way into higher impact journals. Each of the advanced undergraduate team leaders still took a great deal of ownership of their portion of these projects and was responsible for writing up the early drafts of their part of the study. However, more of these eventually became merged into larger manuscripts which provided much stronger, multiexperiment papers, resulting in more undergraduate co-authorships, albeit with only a few undergraduates becoming first authors (Table 1).

**Table 1:** Numbe of publications involving undergraduate students in the lab of the author from 1987 to 2018.

**Year**	**Number of publications from the lab**	**Number of publications involving undergraduates**	**Number of undergraduate coauthors**	**Number of undergraduate first-authors**
1987–2012	47	11	20	2
2012	7	2	4	0
2013	2	2	6	0
2014	8	4	17	1
2015	8	2	9	0
2016	7	1	4	1
2017	14	5	11	0
2018	13	6	13	0

*The relative number of undergraduate authors increased (as did the relative quality of publication) with the addition of senior graduate students in the lab of the author (by the year 2012), although the relative number of first-author publications by undergraduates remained lower than would be expected if there were no graduate students in the program*.

The proof of the success of this approach became obvious when our undergraduates started winning most of the undergraduate research awards at the annual meetings of the Michigan Chapter of the Society for Neuroscience (at one point winning 8 out of the 10 successive Outstanding Undergraduate Research Awards). This TEAM (together everyone achieves more) approach was the primary basis for CMU winning the Outstanding Undergraduate Program of the Year Award in 2013, given by the Society for Neuroscience (Dunbar, 2015). Our approach utilized this R2 advantage (i.e., graduate student mentors) to build on a similar team approach to research that was utilized by Baldwin-Wallace College, which received the award the preceding year (Mickley et al., 2003).

However, the growth of our program and the university has caused a strain on our ability to continue to increase the proportion of our majors engaged in published research. During the past 4 years, our program has grown to over 200 majors, which, in conjunction with escalating costs for animal care to allow vivarium space for CMU's new medical school, has forced us to drop the requirement that all our majors complete a research project. As a result, less than half of our majors are now engaged in research or belong to a lab at CMU. The cost of supporting undergraduate student research became prohibitively high for many faculty mentors and a greater emphasis on conducting research so that faculty can obtain individual grants rather than focusing on student-centered research has negatively impacted our ability to expand the proportion of undergraduates engaged in publishable research.

## Future Directions

Although my colleagues and I may have maximized our ability to increase the proportion of our majors who are engaged in publishable research in our labs, we continue to focus our efforts on increasing the quality of student research. Utilizing ideas propagated by advocates for “open science” (e.g., Yong, 2012; Cummings and Calin-Jageman, 2017) who emphasize reproducibility and transparency, we are now initiating new standards for students in our lab to register their research proposals at the Open Science Framework (http://help.osf.io/m/registrations/l/524205-registeryour-project). In this way, we are committing ourselves to full disclosure of what measures we will be using and how they will be analyzed. This will ensure that all our data and the analyses we employed will be available and will minimize the temptation to “cherry pick” positive results for publication. Our hope is that this increased rigor will translate into more impactful research articles authored or co-authored by our undergraduates.

## Conclusions

A peer-mentoring, team-approach to involving students in publishable research can be very effective and may be augmented by the addition of graduate students as team leaders in R2 universities. The strategies we have employed over the years have proven to be very effective in maximizing the involvement of undergraduates, while increasing the quantity and quality of our published research. However, the advantages of employing this at a growing R2 university, which requires an expanded infrastructure and the support of graduate students, necessitates a delicate balance between focusing on faculty-driven research that is geared toward garnering grant money and concentrating on student-centered research, which prioritizes nurturing students and immersing them in publishable research. Importantly, we are committed to increasing the quality of undergraduate research and by ensuring our students register their proposals, we will be taking another critical step in mentoring students to produce high-quality, transparent publications.

## Author Contributions

The author confirms being the sole contributor of this work and has approved it for publication.

### Conflict of Interest Statement

The author declares that the research was conducted in the absence of any commercial or financial relationships that could be construed as a potential conflict of interest.
